# Association Between Exposure to Air Pollutant Mixture and Risk of Inflammatory Bowel Disease: Modifying Effects of Healthy Lifestyle and Residential Greenspace

**DOI:** 10.3390/toxics14040333

**Published:** 2026-04-16

**Authors:** Runze Bai, Xiaochi Zhang, Guoao Li, Yiyi Wang, Hujia Zhang, Baopeng Liu, Xiuli Zuo, Jie Yan, Qi Zhao

**Affiliations:** 1 Department of Gastroenterology, Qilu Hospital of Shandong University, Jinan 250012, China; brz0713@163.com (R.B.); zuoxiuli@sdu.edu.cn (X.Z.); 2 Shandong Provincial Clinical Research Center for Digestive Disease, Jinan 250012, China; 3 Laboratory of Translational Gastroenterology, Qilu Hospital of Shandong University, Jinan 250012, China; 4 Robot Engineering Laboratory for Precise Diagnosis and Therapy of GI Tumor, Qilu Hospital of Shandong University, Jinan 250012, China; 5 Department of Epidemiology, School of Public Health, Cheeloo College of Medicine, Shandong University, Jinan 250012, China; zhangxiaochi@mail.sdu.edu.cn (X.Z.); guoaoli@mail.sdu.edu.cn (G.L.); 15854890533@163.com (Y.W.); zhanghujiaac@163.com (H.Z.); baopeng.liu@sdu.edu.cn (B.L.); 6 Advanced Medical Research Institute, Cheeloo College of Medicine, Shandong University, Jinan 250012, China

**Keywords:** inflammatory bowel disease, air pollutant mixture, effect modification

## Abstract

**Background:** Although air pollution is increasingly considered an environmental hazard for inflammatory bowel disease (IBD), existing evidence predominantly relies on single-pollutant models that fail to capture mixed exposures, with modifying effects of individual lifestyle and residential environments remaining largely unexplored. **Methods:** We conducted a prospective cohort study using UK Biobank data, including 323,608 participants followed for incident IBD. Annual mean concentrations of five air pollutants [nitrogen dioxide (NO_2_), nitrogen oxides (NO_x_), and PM with aerodynamic diameters of ≤2.5, 2.5–10, and ≤10 μm (PM_2.5_, PM_2.5–10_, PM_10_)] and greenspace percentage within 300 m and 1000 m buffers were assigned to each participant’s residential address. A healthy lifestyle score was defined by five factors: smoking status, alcohol consumption, physical activity, sleep patterns, and dietary quality. Cox proportional hazards models with quantile g-computation (QGC) were employed to examine associations between single- and mixed-air-pollutant exposures and IBD risk. Stratified analyses were performed by healthy lifestyle, lifestyle score, and greenspace percentage. **Results:** During the follow-up period, 1649 and 805 participants developed ulcerative colitis (UC) and Crohn’s disease (CD), respectively. Single-pollutant models suggested that exposures to most air pollutants were substantially associated with increased risk of IBD, and the association strengths were more pronounced for UC than for CD. QGC analyses indicated that the hazard ratios (*HR*) of IBD risk were 1.068 (95%*CI*: 1.018–1.121) for each one-quantile increase in the air pollutant mixture, with NO_2_ weighted as the largest contributor. High physical activity was significantly linked to an attenuated UC-pollutant mixture association. **Conclusions:** This study found that exposure to an air pollutant mixture was associated with increased risk of IBD, especially for UC, with NO_2_ contributing the largest effect size. The certain attenuated air pollution effects of healthy lifestyles and residential greenspaces underscore the need for integrated public health strategies with environmental management.

## 1. Introduction

Inflammatory bowel disease (IBD), including Crohn’s disease (CD) and ulcerative colitis (UC), is a chronic immune-mediated disorder characterized by recurrent gastrointestinal inflammation and a significant systemic burden [1]. As an incurable, relapsing-remitting condition, it inflicts a lifelong toll of physical disability and psychological morbidity on patients [2]. While the current IBD prevalence in developed countries in North America and Europe has surpassed 0.3%, the burden in newly industrialized nations across Asia, South America, and Africa is rising, where lifestyles and environments are becoming increasingly westernized [3]. Namely, IBD has been evolving into a global epidemic and therefore threatens the long-term sustainability of modern healthcare systems [4]. In this context, exploring the key drivers of IBD incidence is necessary for developing more efficient health promotion strategies.

Current evidence indicates that IBD arises from interactions among genetic susceptibility, immune dysfunction, and environmental exposures [5]. However, the incidence surge in certain industrializing countries over a relatively short period could not reasonably be explained by genetic factors alone, which has shifted preventive attention towards modifiable environmental determinants [6]. Among these, ambient air pollution has emerged as a plausible risk factor, considering its linkage to systemic inflammation, oxidative stress, metabolic disruption, and host-microbiome interactions [7,8]. Previously, several epidemiological studies have explored associations between the incidence of IBD and long-term exposure to ambient air pollutants, e.g., particulate matter (PM) and nitrogen oxides (NO_x_) [9,10]. However, existing findings remain inconsistent. One key issue is that the single-pollutant models widely applied by these studies fail to capture the real-world exposure patterns in which pollutants co-occur and may act jointly [11]. Mixed exposures may produce synergistic, antagonistic, or additive health effects [12], and methods that estimate the health impact of air pollutant mixtures may therefore provide a more realistic assessment of risk. To date, this mixture modeling framework, such as quantile g-computation (QGC), has not been widely applied in IBD epidemiology, leaving an important gap in the evidence base.

In addition to environmental exposures, individual and neighborhood-level factors may modify the adverse health effects of air pollution exposure [13,14]. For example, a large amount of evidence has shown that healthy lifestyle behaviors such as non-smoking, limited alcohol consumption, a balanced diet, regular physical activity, and adequate sleep are associated with reduced systemic inflammation and immune function [15,16,17]. Potential mechanisms include improved antioxidant capacity, gut microbiota stability, and others [18,19]. Likewise, residential greenspaces may attenuate the pollution-related risk of IBD [20]. The underlying pathways may include improving local air quality and reducing stress [21]. Despite growing evidence on these factors individually, their potential roles in modifying the association between mixed-air-pollution exposure and IBD have not been well characterized. Addressing this uncertainty is essential for refining IBD prevention frameworks and public health interventions.

To address these knowledge gaps, this study examined the association between long-term exposure to an air pollutant mixture and incident IBD using UK Biobank data and further assessed whether healthy lifestyle and residential greenspace might provide certain modifying effects.

## 2. Methods

### 2.1. Study Population

**Figure 1:**
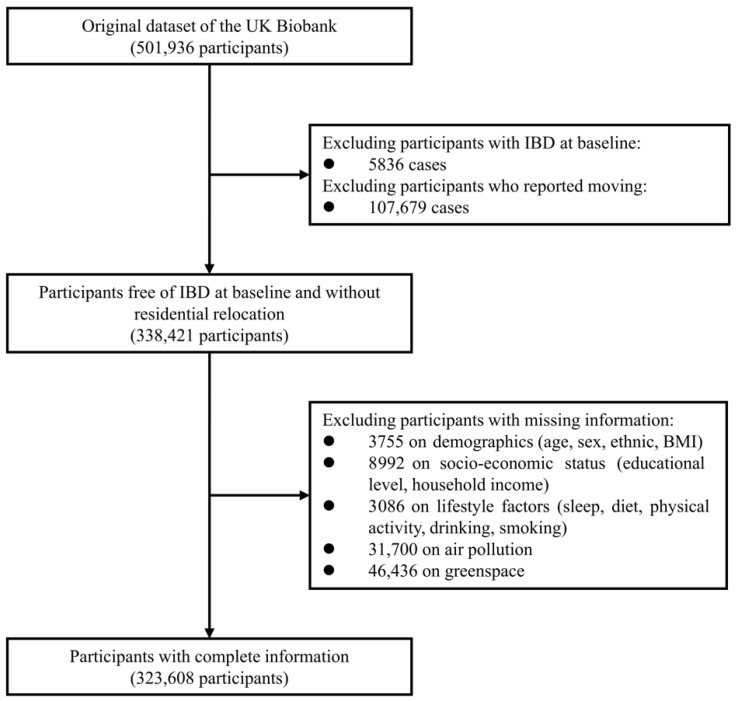
Flow chart of participant inclusion and exclusion.

We conducted a prospective cohort study using data from the UK Biobank (application number 91536), a large population-based cohort study enrolling over 500,000 adults aged 40–69 years across the UK between 2006 and 2010. Detailed phenotypic and genotypic information was collected via baseline questionnaires, physical measurements, biological sampling, and longitudinal follow-up of various health outcomes. The study protocol was approved by the UK North West Multicenter Research Ethics Committee, and all participants provided written informed consent prior to their inclusion. As illustrated in Figure 1, we excluded participants diagnosed with IBD at baseline, those who reported moving residence after recruitment, and those with missing data on environmental exposures and covariates.

### 2.2. Environmental Exposure Measurements

Annual mean concentrations of nitrogen dioxide (NO_2_), NO_x_, and PM with aerodynamic diameters of ≤2.5, 2.5–10, and ≤10 μm (PM_2.5_, PM_2.5–10_, PM_10_) were estimated for each residential address of participants using validated land use regression (LUR) models, as previously described [22,23]. Briefly, the LUR models were developed based on standardized monitoring campaigns across 20 European study areas, with GIS-derived predictor variables including traffic intensity, population density, and land use. The cross-validation R^2^ for PM_2.5_, PM_2.5–10_, PM_10_, NO_2_, and NO_x_ was 77%, 57%, 88%, 87%, and 88%, respectively, in the southeast England area (London/Oxford), indicating good model performance across all pollutant metrics included in our study.

Following previous UK Biobank studies, residential greenspace percentage was estimated around each participant’s baseline address using the Generalized Land Use Database for England [24,25,26]. Greenspace percentage was calculated within predefined residential buffers (e.g., 300 m and 1000 m) and then categorized into three levels: high (the highest quintile), intermediate (quintiles 2–4), and low (the lowest quintile).

### 2.3. Ascertainment of Outcomes and Covariates

The primary health outcome was incident IBD throughout the follow-up period, which was ascertained via linkage to the National Health Service (NHS) databases, including hospital admission records, primary care data, and cancer and death registry data. The follow-up period extended from participant enrollment (baseline) to 31 March 2023. IBD subtypes were coded using the International Classification of Diseases, version 10 (ICD-10), with K50 for CD and K51 for UC.

The following covariates collected at baseline were included considering their potential confounding effects: age at recruitment, sex, ethnicity (white or other), educational level (college degree or other), average total household income before tax (<£18,000, £18,000–£100,000, >£100,000, or unknown), employment status (paid job, unpaid job, or retired), body mass index (BMI) [categorized according to World Health Organization (WHO) criteria as underweight (<18.5 kg/m^2^), normal weight (18.5 ≤ BMI < 25 kg/m^2^), overweight (25 ≤ BMI < 30 kg/m^2^), and obese (≥30 kg/m^2^)], and average time spent outdoors (hours/day).

Furthermore, five lifestyle-related factors were incorporated into a lifestyle score: smoking and alcohol consumption status, sleep patterns, physical activity, and adherence to an IBD-associated healthy diet. For example, a score of five was defined as never smoking, never drinking, optimal sleep, adequate physical activity, and high dietary quality. Detailed definitions of these five factors were provided in Supplementary Text S1. Participants were classified by their cumulative score (range: 0–5) into unhealthy (<3) and healthy (≥3) lifestyle groups for stratified analyses [16].

### 2.4. Statistical Analysis

Cox proportional hazards models were used to quantify the association between each air pollutant and the risk of IBD incidence, with the effect size described as hazard ratios (*HR*s) and 95% confidence intervals (*CI*s). Our main multivariable model was adjusted for age at recruitment, sex, ethnicity, educational level, household income, BMI, employment status, time spent outdoors, and a healthy lifestyle status. Furthermore, QGC was applied for survival outcomes to evaluate the joint effect of multi-pollutant exposure (i.e., PM_2.5_, PM_10_, PM_2.5–10_, NO_2,_ and NO_x_). The QGC approach commonly estimates the overall change in disease risk associated with a one-quantile simultaneous increase in the air pollutant mixture and provides weights for the relative contribution of each component [12]. Stratified analyses were conducted by healthy lifestyle status, lifestyle score, and residential greenspace percentage to explore their potential modifying effects on the effect sizes of single- and mixed-air-pollution exposure. Interaction models were constructed to examine the significance of effect modification by healthy lifestyle and greenspace percentage.

Sensitivity analyses were performed to evaluate the robustness of the main findings under alternative model specifications and analytic restrictions. For both single-pollutant and pollutant mixture models, a simplified model was constructed as a comparator to the main model. The simplified models were adjusted only for age at recruitment, sex, ethnicity, and average time spent outdoors in summer and winter. The proportional hazards assumption was verified for all Cox models using Schoenfeld residuals, and relative model fits were compared across specifications using the Akaike Information Criterion (AIC). Differences between simplified and main models were tested using a Z-test [27], with two-sided *p* values (*P_diff_*) < 0.05 considered statistically significant.

## 3. Results

### 3.1. Descriptive Results

In this study, 323,608 participants at baseline [average age with standard deviation (SD): 57.6 ± 8.0 years] were included, with 1649 and 805 cases developing UC and CD during follow-up, respectively. The study population was predominantly female (54.3%), of White ethnicity (91.2%), in paid employment (56.0%), with an annual household income of £18,000–100,000 (61.6%), and did not hold a college degree (68.9%). In addition, 42.6% of participants were overweight, with 51.2% classified as having a healthy lifestyle. The mean concentrations of NO_x_, NO_2_, PM_2.5,_ PM_10_, and PM_2.5–10_ with SD were 43.9 ± 15.2, 26.6 ± 7.5, 10.0 ± 1.0, 16.2 ± 1.9, and 6.4 ± 0.9 µg/m^3^, respectively. Mean residential greenspace coverage was 35.4 ± 23.0% within a 300 m buffer and 45.4 ± 21.4% within a 1000 m buffer (Table 1).

### 3.2. Associations Between Air Pollution Exposure and Risk of IBD Incidence

Results of single-pollutant models suggested that exposure to each air pollutant except for PM_10_ and PM_2.5–10_ was significantly associated with increased risk of IBD (Table S1). For example, the *HR* of IBD incidence was 1.088 (95%*CI*: 1.032–1.147) per IQR rise in NO_2_. Type-specific analyses indicated that all five air pollutants were positively associated with increased risk of UC incidence, while no air pollution exposure was significantly linked to CD risk.

**Figure 2:**
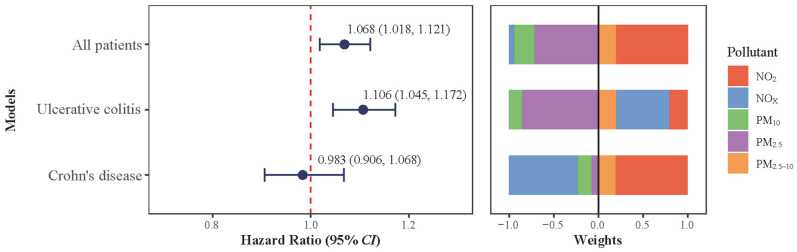
Associations between mixed-air-pollutant exposure and inflammatory bowel disease and its subtypes. UC: ulcerative colitis; CD: Crohn’s disease; NO_2_: nitrogen dioxide; NOx: nitrogen oxides. PM_2.5_: particulate matter with aerodynamic diameters of ≤2.5 μm. PM_10_: particulate matter with aerodynamic diameters of ≤10 μm. PM_2.5–10_: particulate matter with aerodynamic diameters of 2.5–10 μm. Models were adjusted for age at recruitment, sex, ethnicity, educational level, average total household income before tax, body mass index, employment status, average time spent outdoors in summer and winter, and healthy lifestyle (categorized into healthy and unhealthy groups).

Results of QGC analyses suggested that the *HR* of IBD incidence was 1.068 (95%*CI*: 1.018–1.121) for each one-quantile increase in the air pollutant mixture (Figure 2), with NO_2_ contributing to the largest positive weight, followed by PM_2.5_ and PM_2.5–10_. Type-specifically, results of mixture analysis aligned with the single-pollutant findings for UC, such that the *HR* was 1.106 (95%*CI*: 1.045–1.172) for a one quantile increase in the air pollutant mixture. The weights for the UC model differed slightly from the overall QGC model, with NO_x_ showing substantial positive contributions alongside NO_2_ and PM_2.5_. In comparison, no significant association was observed between the air pollutant mixture and CD (*HR* = 0.983, 95%*CI*: 0.906–1.068).

### 3.3. Effect Modification by Healthy Lifestyle and Greenspace

**Figure 3:**
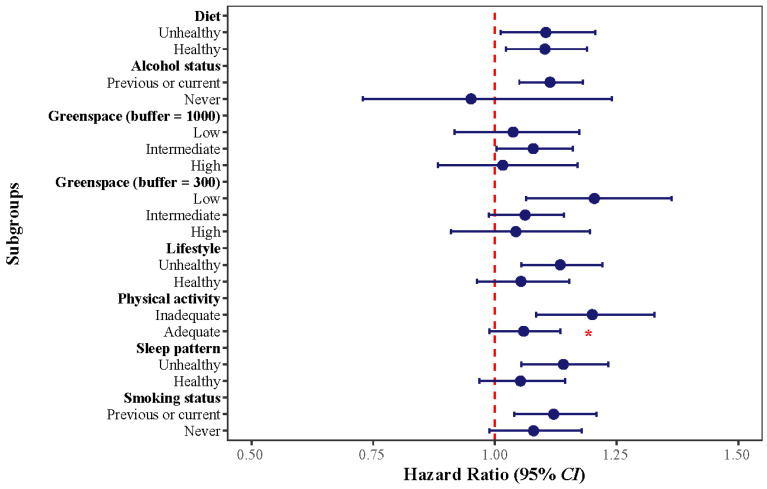
Effect modification by lifestyle factors and residential greenspace on the association between mixed-air-pollutant exposure and UC. *CI*: confidence interval. * denotes *p* value for the following interaction: * *P_int_* < 0.05.

Results of both the single-pollutant models and QCG analyses suggested that physical activity was a significant effect modifier for the association between air pollution exposure and UC (Figure 3 and Figures S1–S5). *HR*s were 1.200 (95%*CI*: 1.085–1.328) and 1.059 (95%*CI*: 0.988–1.135) per one-quantile increase in the air pollutant mixture for participants with inadequate and adequate physical activity, respectively (*P_int_* = 0.037). For other lifestyle factors, the adverse effects of the air pollution mixture were also only pronounced in high-risk groups, although heterogeneities were not statistically significant in comparison to the low-risk groups. Again, the associations between each single air pollutant and air pollutant mixture with IBD risk were only significant for the group with a low composite healthy lifestyle score (*HR* = 1.134; 95%*CI*: 1.054–1.221), compared to the group with the highest score (*HR* = 1.054; 95%*CI*: 0.963–1.153), though the difference was not statistically significant (*P_int_* = 0.334).

Regarding residential greenspace percentage, its modifying effect was more discernible within the 300 m buffer zone: participants living in low greenspace areas experienced the highest risk of IBD from air pollution mixture exposure (*HR* = 1.205; 95%*CI*: 1.065–1.363). This risk was numerically higher than that observed in areas with intermediate (*HR* = 1.062; 95%*CI*: 0.988–1.143) and high (*HR* = 1.043; 95%*CI*: 0.910–1.195) greenspace levels.

Sensitivity analyses demonstrated that the results of the main models consistently achieved superior fit compared to the simplified models across all exposure specifications, as evidenced by uniformly lower AIC values, with no statistically significant differences in effect estimates observed between the two model specifications for any single-pollutant or the mixture, indicating the robustness of the main findings (Table S2).

## 4. Discussion

In this large population-based study, we observed that long-term exposure to air pollution was linked to a higher risk of IBD incidence, with the strength of association more pronounced for UC than for CD. The estimated mixture effect of air pollutants was larger than most single-pollutant estimates, and traffic-related pollutants, particularly NO_2_ and PM_2.5_, were identified as the dominant contributors. Furthermore, potentially modifiable factors appeared to shape susceptibility, such that the adverse effects of air pollution were substantially attenuated or insignificant among participants with adequate physical activity, those residing in areas with higher greenspace coverage, and those with healthy lifestyles.

### 4.1. Principal Findings and Comparison with Previous Studies

Our finding that exposure to traffic-related pollutants might be associated with increased risk of IBD aligns with previous UK Biobank analyses [9,10,28], as well as studies in other settings. For example, outpatient visits for UC have been shown to increase with rising PM_2.5_ concentrations in China [29]. At the same time, heterogeneous evidence exists, with certain multicenter European studies reporting significant associations with total traffic burden but null results for individual pollutants [30]. This inter-study inconsistency may be partly explained by limited sample size and differences in exposure characterization (e.g., land-use regression versus satellite-chemical transport hybrids), case definitions and ascertainment, and follow-up duration. More importantly, findings from many early studies relied primarily on single-pollutant models, which can be difficult to interpret when pollutants share sources and are strongly correlated [31].

In comparison to previous studies, our mixture framework was designed to estimate the joint impact of concurrent exposures while retaining interpretability through component weights. First, we observed that the associations between air pollution exposure and risk of IBD incidence were only significant for UC. This result may partly reflect etiologic differences between these two IBD subtypes. UC primarily affects the colonic mucosa with a continuous inflammation pattern [32], whereas CD can involve any gastrointestinal segment and is typified by transmural inflammation and skip lesions [1]. It is speculated that the etiologic window may be more readily detectable for UC, considering that pollution-related injury operates predominantly through systemic inflammatory activation, oxidative stress, or perturbation of the colonic mucosal barrier [33]. Additionally, the gut microbiome composition, which differs substantially between UC and CD patients, may respond differentially to environmental stressors introduced via air pollution exposure [34,35].

In addition, our analysis revealed that the overall effect of the air pollutant mixture exceeded most individual pollutant effects. This finding is compatible with additive or synergistic toxicity in real-world exposure settings, where individuals are concurrently exposed to a series of traffic- and combustion-related pollutants. Notably, NO_2_ and NO_x_ dominated this elevated risk. Mechanistically, the observed synergistic toxicity of the air pollutant mixture likely stems from the intricate physicochemical coupling and biological interactions among its components. NO_2_ and NO_x_ are essential precursors in photochemical reactions to form secondary inorganic and organic aerosols, thereby directly contributing to the chemical complexity and toxicity of PM_2.5_ [36]. PM can carry adsorbed gaseous pollutants and other combustion by-products, facilitating their transport and localized delivery to the gastrointestinal mucosa [37]. Experimental studies further provide supportive evidence that PM exposure could cause intestinal barrier injury by disrupting tight junctions, depleting systemic antioxidant reserves, and disrupting microbiota composition equilibrium [38,39]. This compromised barrier integrity renders the colonic mucosa hypersensitive to the inflammatory insults delivered by the gaseous pollutants, resulting in a cumulative pathogenic effect that significantly exceeds that of single-pollutant exposure. Finally, the preponderant weights of NO_2_ and NO_x_ in our mixture model not only highlight the specific relevance of traffic-related emissions to IBD pathogenesis but also underscore the critical need to evaluate multi-pollutant exposures rather than relying exclusively on single-pollutant paradigms in environmental health assessments [40].

### 4.2. Effect Modification by Lifestyle and Greenspace

The attenuation of pollution-IBD associations among physically active participants is consistent with previous reports that behavioral factors may shape the host response to environmental exposures. There are several mechanisms. Regular physical activity has well-described anti-inflammatory effects and may enhance antioxidant defense capacity [41], potentially counterbalancing pollutant-induced oxidative stress and systemic inflammation [42]. Experimental evidence demonstrates that exercise beneficially modulates favorable gut microbial profiles through enrichment of butyrate-producing taxa and the downregulation of colonic pro-inflammatory cytokines, thereby priming an overall anti-inflammatory status [43]. Additionally, physical activity may influence the body’s handling of inhaled pollutants through effects on pulmonary function, mucociliary clearance, and metabolic capacity for xenobiotic detoxification [44]. Beyond physical activity, our findings indicate a broader modulatory role for the overall lifestyle matrix. While the protective trends of optimal diet, sleep, and smoking abstention did not reach statistical significance, deleterious habits such as alcohol consumption notably exacerbated the effect size of NO_x_ exposure. Future studies integrating exposome frameworks and multi-level lifestyle assessments may help disentangle the complex interplay between environmental exposures and behavioral factors in IBD etiology.

The differential modifying effects observed across buffer sizes provide insights into the spatial scale at which greenspace confers protection against air pollution exposure. In comparison to the 1000 m buffer, the only pronounced modifying effect within the 300 m buffer suggests that access to greenspace in residential microenvironments within walking distance may be more relevant to health protection than broader neighborhood greenspace availability [45]. However, for the air pollutant mixture exposure, the modifying effect of greenspace did not reach conventional statistical significance, although it maintained a trend consistent with the single-pollutant models. This may reflect the limited capacity of greenspace to mitigate adverse effects when multiple pollutants act synergistically, especially in urban areas [21]. Existing evidence speculates that greenspace may reduce IBD risk related to air pollution exposure through several complementary psychosocial and behavioral mechanisms: (1) promoting physical activity, which enhances anti-inflammatory responses, strengthens intestinal barrier function, and beneficially modulates the gut microbiome [41,46,47]; (2) reducing psychological stress through attention restoration and cortisol reduction, thereby positively regulating the gut–brain axis and decreasing stress-induced intestinal inflammation and permeability [48,49]; (3) affecting air quality by particle deposition, dispersion, and modification [50]; (4) creating physical barriers that increase distance between traffic emissions and residential areas, reducing personal exposure even when ambient concentrations remain elevated [50]. However, further well-designed epidemiological and experimental studies are highly encouraged to explore these hypotheses.

### 4.3. Public Health and Clinical Implications

Although our observational findings require cautious interpretation, several implications warrant discussion. These findings should be interpreted in the context of long-term improvements in UK air quality. According to official statistics published by the UK Department for Environment, Food and Rural Affairs (DEFRA), total NO_x_ emissions in the UK decreased by 80% between 1990 and 2024, with road transport NO_x_ emissions declining by 73% between 2005 and 2024, driven by stricter vehicle emission standards and the uptake of electric vehicles [40]. Over the same period, PM_2.5_ emissions fell by 46% [51]. Despite these substantial improvements, air pollution remains a persistent public health concern, such that in 2024 the annual mean NO_2_ concentration in numerous roadside locations across UK urban areas remained higher than the national limit value [40]. Crucially, our finding that NO_2_ contributed the largest effect of the air pollutant mixture suggests that even at the relatively low exposure level, traffic-related air pollution may continue to exert a meaningful influence on chronic gastrointestinal disease risk. At the population level, these results suggest the importance of multi-pollutant mitigation strategies, particularly those targeting traffic-related emissions, in efforts to prevent or reduce the incidence of UC. At the community level, urban planning strategies that increase access to greenspace may yield co-benefits, especially in close proximity to residences. At the individual level, maintaining regular physical activity may be a pragmatic strategy for disease prevention and control, particularly for populations living in highly polluted areas.

### 4.4. Strengths and Limitations

This study has several strengths. First, the prospective cohort design with a large sample size and a comprehensive assessment of environmental exposures and lifestyle factors enabled robust analysis of complex exposure-outcome relationships. Second, the use of QGC analyses allowed estimation of the joint effects of correlated pollutants and summarization of their relative contributions within the mixture, complementing traditional single-pollutant models. Third, separate analyses for UC and CD facilitated identification of subtype-specific patterns that could be missed in aggregated IBD analyses. Finally, the assessment of modifying effects across multiple lifestyle factors and spatial scales of greenspace exposure provides a more comprehensive understanding of environmental susceptibility to IBD. However, several limitations should be considered. Exposure estimates were assigned based on residential addresses, which may not fully capture personal exposure given time spent away from home (e.g., work, commuting). Such non-differential exposure misclassification would generally bias associations toward the null, suggesting that our findings may underestimate true effects. Additionally, greenspace percentage captured overall land cover exposure, while the lack of data on greenspace quality and type might affect health outcomes. Despite adjustment for a series of socioeconomic factors, i.e., household income, educational attainment, and employment status, it is still possible that the residual confounding by unmeasured or imprecisely captured factors may not be entirely excluded. Future studies are encouraged to incorporate more granular socioeconomic measures. Finally, the specific biological pathways linking air pollution to IBD risk remain incompletely understood from epidemiological evidence.

## 5. Conclusions

In this large prospective cohort study, exposure to mixed air pollution was associated with increased risk of IBD, especially for its subtype UC. Integrating stringent emission controls with the promotion of healthy lifestyles and urban greenspace represents an actionable public health strategy to mitigate the burden of IBD related to environmental susceptibility.

## Figures and Tables

**Table 1:** Baseline characteristics for IBD patients.

Variable	Overall (n = 323,608)	Subsequent UC (n = 1649)	Subsequent CD (n = 805)
**Age at baseline (mean (SD))**	57.59 (8.0)	58.59 (7.9)	57.89 (8.1)
**Sex (%)**			
Female	175,646 (54.3)	785 (47.6)	449 (55.8)
Male	147,962 (45.7)	864 (52.4)	356 (44.2)
**Household Income (%)**			
<£18,000	64,579 (20.0)	388 (23.5)	217 (27.0)
£18,000–£100,000	199,446 (61.6)	951 (57.7)	427 (53.0)
>£100,000	13,960 (4.3)	44 (2.7)	21 (2.6)
unknown	45,623 (14.1)	266 (16.1)	140 (17.4)
**Education (%)**			
College	100,566 (31.1)	407 (24.7)	200 (24.8)
Others	223,042 (68.9)	1242 (75.3)	605 (75.2)
**Ethnicity (%)**			
White	295,274 (91.2)	1491 (90.4)	752 (93.4)
Others	28,334 (8.8)	158 (9.6)	53 (6.6)
**BMI (%)**			
Underweight	1714 (0.5)	6 (0.4)	6 (0.7)
Normal	105,000 (32.4)	465 (28.2)	230 (28.6)
Overweight	137,976 (42.6)	732 (44.4)	324 (40.2)
Obese	78,918 (24.4)	446 (27.0)	245 (30.4)
**Employment (%)**			
Paid job	181,172 (56.0)	832 (50.5)	398 (49.4)
Retired	116,647 (36.0)	660 (40.0)	316 (39.3)
Unpaid job	25,789 (8.0)	157 (9.5)	91 (11.3)
**Physical activity (%)**			
Inadequate	89,100 (27.5)	526 (31.9)	257 (31.9)
Adequate	234,508 (72.5)	1123 (68.1)	548 (68.1)
**Smoking status (%)**			
Never	178,095 (55.0)	709 (43.0)	351 (43.6)
Previous or current	145,513 (45.0)	940 (57.0)	454 (56.4)
**Alcohol status (%)**			
Never	14,036 (4.3)	79 (4.8)	53 (6.6)
Previous or current	309,572 (95.7)	1570 (95.2)	752 (93.4)
**Diet (%)**			
Unhealthy	120,028 (37.1)	697 (42.3)	359 (44.6)
Healthy	203,580 (62.9)	952 (57.7)	446 (55.4)
**Sleep pattern (%)**			
Unhealthy	151,615 (46.9)	875 (53.1)	425 (52.8)
Healthy	171,993 (53.1)	774 (46.9)	380 (47.2)
**Lifestyle (%)**			
Unhealthy	157,877 (48.8)	983 (59.6)	476 (59.1)
Healthy	165,731 (51.2)	666 (40.4)	329 (40.9)
**NO_x_** (mean (SD)), µg/m^3^	43.9 (15.2)	45.4 (16.7)	43.83 (15.1)
**NO_2_** (mean (SD)), µg/m^3^	26.6 (7.5)	27.4 (7.9)	26.8 (7.7)
**PM_2.5–10_** (mean (SD)), µg/m^3^	6.4 (0.9)	6.5 (0.9)	6.41 (0.9)
**PM_10_** (mean (SD)), µg/m^3^	16.2 (1.9)	16.4 (1.9)	16.2 (1.9)
**PM_2.5_** (mean (SD)), µg/m^3^	10.0 (1.0)	10.1 (1.1)	10.02 (1.1)
**Greenspace percentage 300 m** (mean (SD)), %	35.4 (23.0)	34.2 (22.1)	35.7 (23.2)
**Greenspace percentage 1000 m** (mean (SD)), %	45.4 (21.4)	44.0 (20.7)	45.0 (21.6)

BMI, body mass index; IBD, inflammatory bowel disease; mean (SD) values and percentages are reported for continuous and categorical variables, respectively.

## Data Availability

The data underlying this article were provided by the UK Biobank. Detailed information about the UK Biobank, including data access policies, can be accessed at https://www.ukbiobank.ac.uk/ (accessed on 5 December 2025).

## Supplementary Materials

The following supporting information can be downloaded at: https://www.mdpi.com/article/10.3390/toxics14040333/s1, Text S1: Definition of a healthy lifestyle; Figure S1: Effect modification by healthy lifestyle and residential greenspace on the association between PM_2.5_ exposure and UC; Figure S2: Effect modification by healthy lifestyle and residential greenspace on the association between NO_2_ exposure and UC; Figure S3: Effect modification by healthy lifestyle and residential greenspace on the association between NO_x_ exposure and UC; Figure S4: Effect modification by healthy lifestyle and residential greenspace on the association between PM_10_ exposure and UC; Figure S5: Effect modification by healthy lifestyle and residential greenspace on the association between PM_2.5–10_ exposure and UC; Table S1: Associations between single air pollutants, greenspace exposure and IBD. Table S2: Sensitivity analysis results for single- and mixed- exposure models [9,52].
