# Supplementary material for: Association Between Exposure to Air Pollutant Mixture and Risk of Inflammatory Bowel Disease: Modifying Effects of Healthy Lifestyle and Residential Greenspace
Source: Toxics. 2026 Apr 16;14(4):333. doi: 10.3390/toxics14040333 (PMC13119546; doi:10.3390/toxics14040333)
Supplement: Supplementary file 1 [file toxics-14-00333-s001.zip › toxics-4201512-supplementary.pdf]

Supplementary Materials

Association between exposure to air pollutant mixture and risk of inflammatory bowel disease: modifying effects of healthy lifestyle and residential greenspace

Table of Contents

Text S1. Definition of a healthy lifestyle.

Figure S1 Effect modification by healthy lifestyle and residential greenspace on the association between $PM_{2.5}$ exposure and UC.

Figure S2 Effect modification by healthy lifestyle and residential greenspace on the association between NO_2 exposure and UC.

Figure S3 Effect modification by healthy lifestyle and residential greenspace on the association between NO_x exposure and UC.

Figure S4 Effect modification by healthy lifestyle and residential greenspace on the association between PM_{10} exposure and UC.

Figure S5 Effect modification by healthy lifestyle and residential greenspace on the association between $PM_{2.5-10}$ exposure and UC.

Table S1. Associations between single air pollutants, greenspace exposure and IBD.

Table S2. Sensitivity analysis results for single- and mixed- exposure models.

Text S1. Definition of a healthy lifestyle

A healthy sleep pattern was defined based on five criteria: (1) being a morning person (morning chronotype); (2) obtaining 7–8 hours of sleep per day; (3) never or rarely/sometimes experiencing insomnia; (4) having no snoring complaints; (5) never or rarely/sometimes experiencing frequent daytime sleepiness[1]. Physical activity was categorized as “Adequate” or “Inadequate” according to American Heart Association recommendations. Adequate activity was defined as ≥ 150 minutes of moderate activity per week, ≥ 75 minutes of vigorous activity per week, an equivalent combination, or moderate physical activity at least 5 days a week. Dietary intake was assessed using a self-reported food frequency questionnaire (FFQ). A healthy diet was defined as consuming at least four of the following seven healthy food groups: (1) fruits ≥ 3 servings/day; (2) vegetables ≥ 3 servings/day; (3) fish ≥ 2 servings/week; (4) processed meats ≤ 1 serving/week; (5) unprocessed red meats ≤ 1.5 servings/week; (6) whole grains ≥ 3 servings/day; and (7) refined grains ≤ 1.5 servings/day. Frequency-based items were converted to quantitative estimates by assigning the mean value of each category (e.g., 2–4 times/week was converted to 3 servings/week)[2].

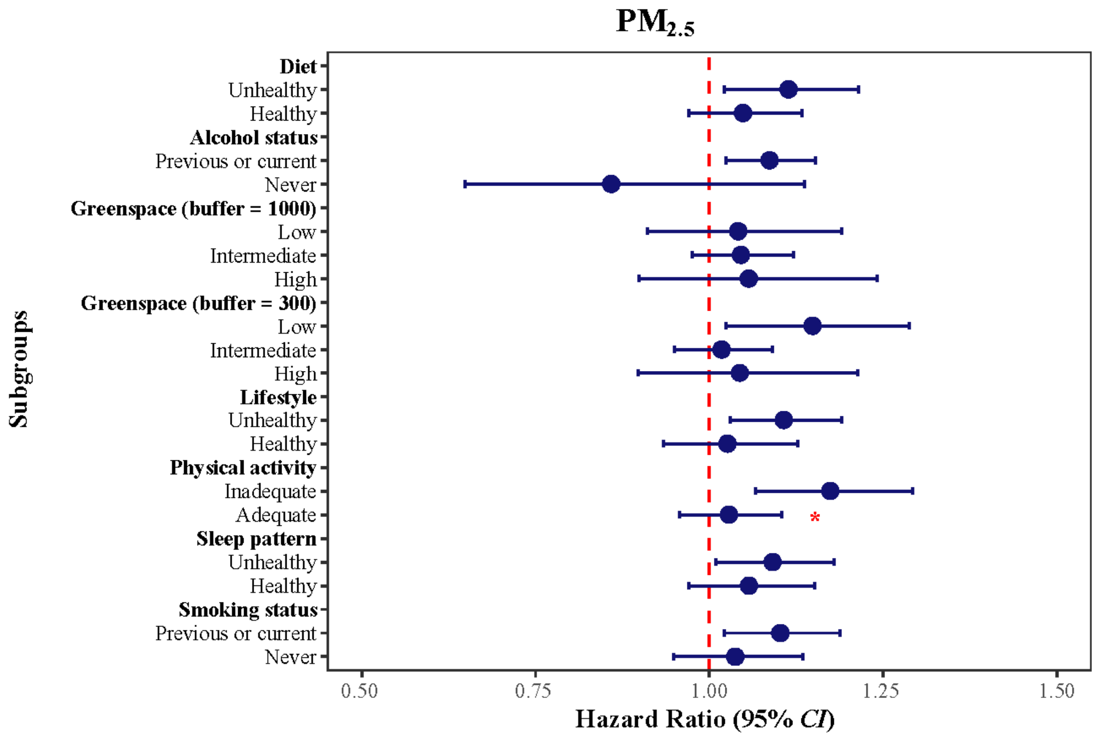

Figure S1. Effect modification by healthy lifestyle and residential greenspace on the association between PM2.5 exposure and UC. CI: confidence interval. * denotes P value for the following interaction: *$P_{int} < 0.05$, ** $P_{int} < 0.01$, *** $P_{int} < 0.001$; ·$P_{int} < 0.1$.

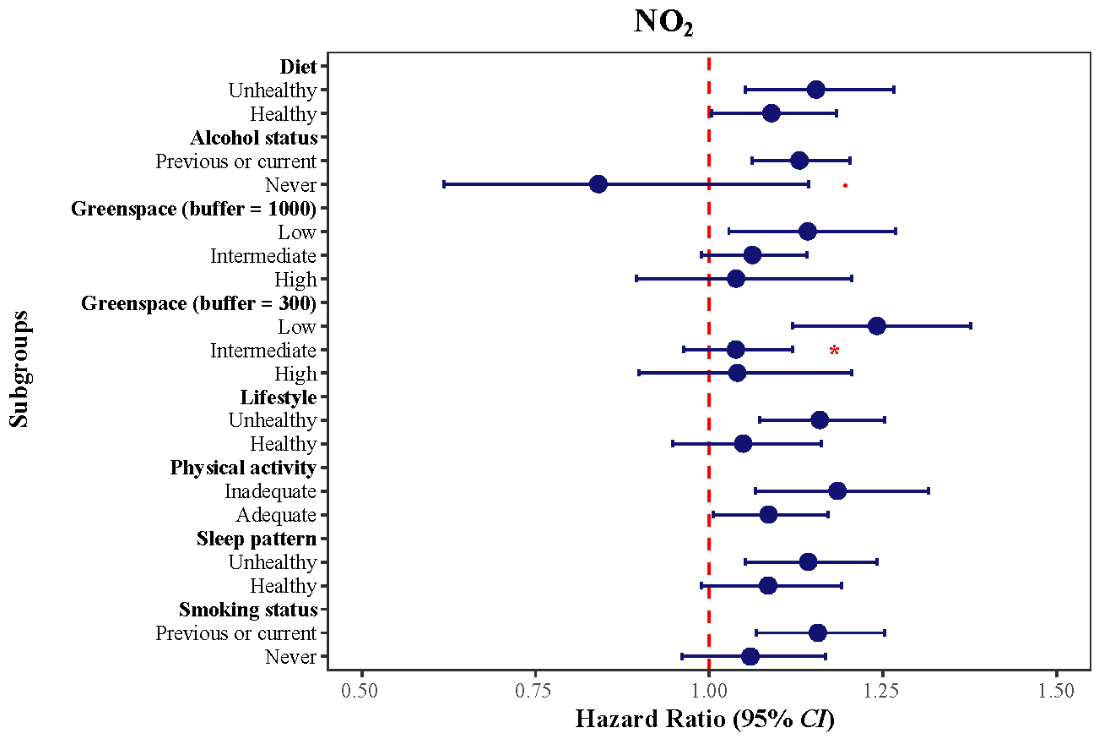

Figure S2. Effect modification by healthy lifestyle and residential greenspace on the association between NO_2 exposure and UC. CI: confidence interval. * denotes P value for the following interaction: *P_{int} < 0.05, ** P_{int} < 0.01, *** P_{int} < 0.001; ·P_{int} < 0.1.

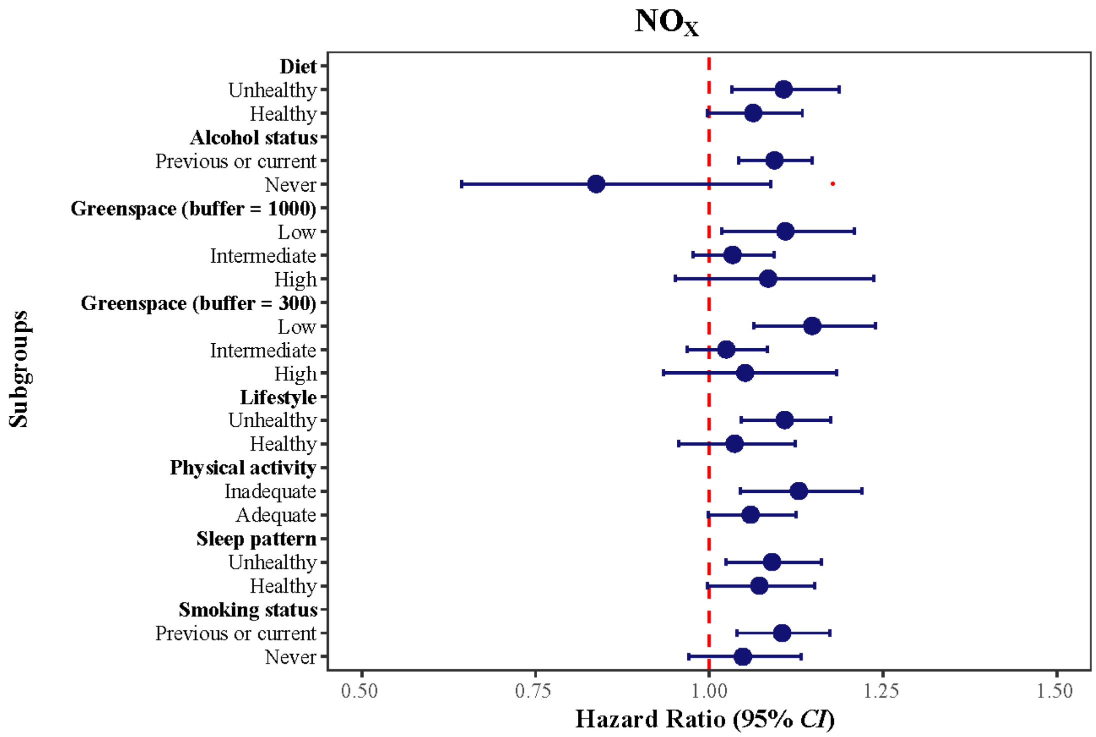

Figure S3. Effect modification by healthy lifestyle and residential greenspace on the association between NOx exposure and UC. CI: confidence interval. * denotes P value for the following interaction: *$P_{int} < 0.05$, ** $P_{int} < 0.01$, *** $P_{int} < 0.001$; ·$P_{int} < 0.1$.

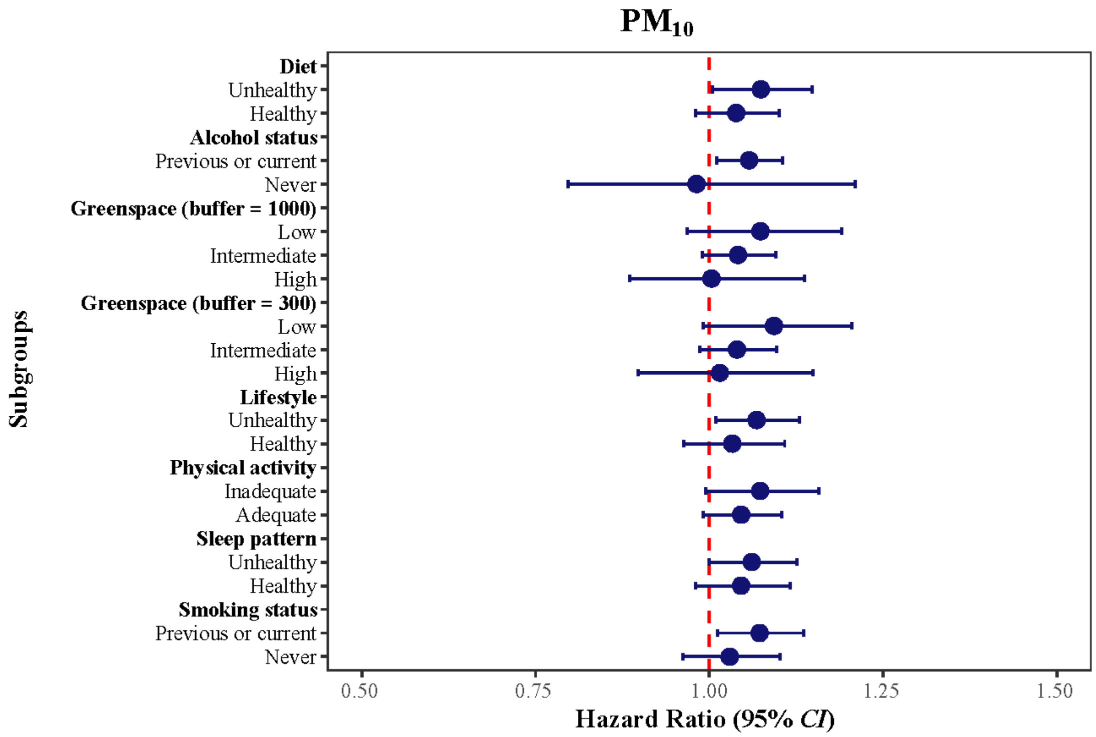

Figure S4. Effect modification by healthy lifestyle and residential greenspace on the association between PM_{10} exposure and UC. *CI*: confidence interval. * denotes P value for the following interaction: *$P_{int} < 0.05$, ** $P_{int} < 0.01$, *** $P_{int} < 0.001$; ·$P_{int} < 0.1$.

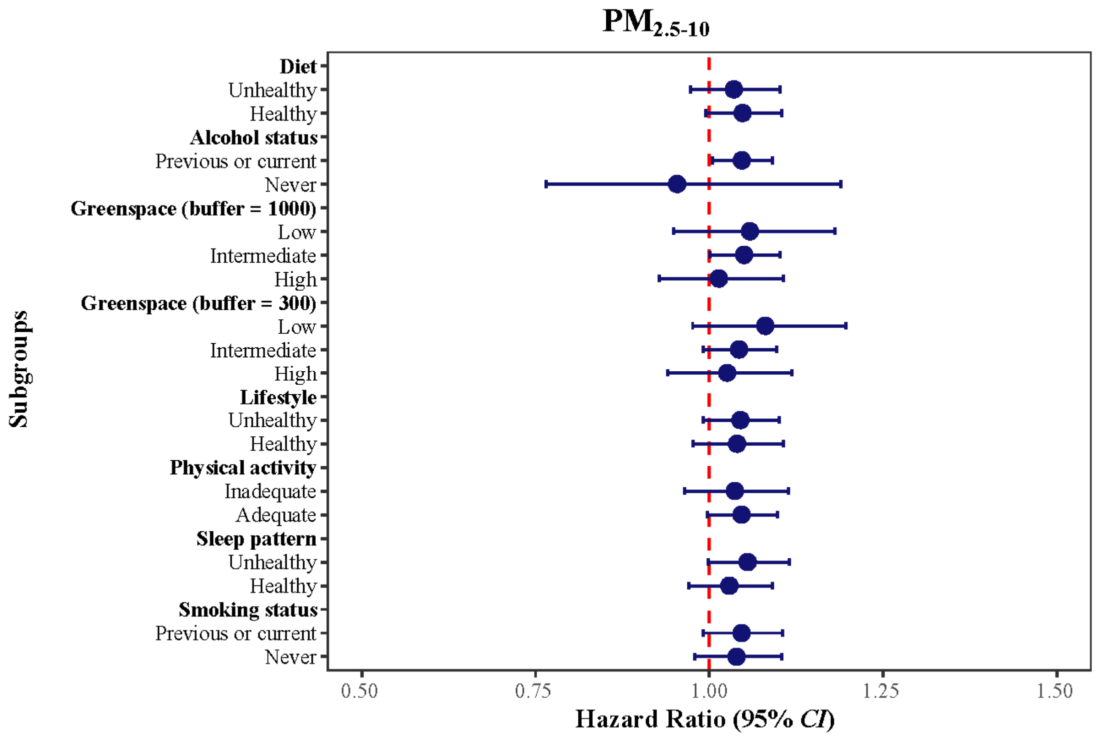

Figure S5. Effect modification by healthy lifestyle and residential greenspace on the association between $PM_{10\text{-}2.5}$ exposure and UC. *CI*: confidence interval. * denotes P value for the following interaction: *P_{int} < 0.05, ** P_{int} < 0.01, *** P_{int} < 0.001; ·P_{int} < 0.1.

Table S1. Associations between single air pollutants, greenspace exposure, and IBD.

**Variable**	**All patients**		**Ulcerative colitis**		**Crohn's disease**	
	HR (95%*CI*)	*p*	*HR* (95%*CI*)	*p*	*HR* (95%*CI*)	*p*
NO_2	1.088 (1.032, 1.147)	0.002	1.117 (1.050, 1.188)	<0.001	0.994 (0.906, 1.090)	0.894
No_x	1.052 (1.009, 1.097)	0.018	1.083 (1.032, 1.135)	0.001	0.959 (0.887, 1.036)	0.287
$PM_{2.5}$	1.059 (1.008, 1.112)	0.023	1.076 (1.015, 1.140)	0.013	0.988 (0.907, 1.075)	0.774
PM_{10}	1.025 (0.987, 1.064)	0.205	1.054 (1.009, 1.101)	0.018	0.962 (0.901, 1.027)	0.245
$PM_{2.5\text{-}10}$	1.024 (0.988, 1.060)	0.189	1.044 (1.002, 1.087)	0.039	0.993 (0.934, 1.055)	0.812

HR: hazard ratio; CI: confidence interval; NO_2: nitrogen dioxide; NOx: nitrogen oxides; $PM_{2.5}$: particulate matter with aerodynamic diameters of ≤2.5 µm. PM_{10}: particulate matter with aerodynamic diameters of ≤10 µm. $PM_{2.5\text{-}10}$: particulate matter with aerodynamic diameters of 2.5–10 µm. Models adjusted for age at recruitment, sex, ethnicity, educational level, average total household income before tax, body mass index, employment status, average time spent outdoors in summer and winter, and healthy lifestyle (categorized into healthy and unhealthy groups).

Table S2. Sensitivity analysis results for single and mixed exposure models.

Variable	Model	*HR* (95%*CI*)	*AIC*	P_{ph}	P_{diff}
$PM_{2.5\text{-}10}$	Main model	1.024 (0.988, 1.060)	57545.57	0.722	ref.
$PM_{2.5\text{-}10}$	Simplified model	1.031 (0.995, 1.067)	57760.01	0.350	0.786
$PM_{2.5}$	Main model	1.059 (1.008, 1.112)	57542.16	0.710	ref.
$PM_{2.5}$	Simplified model	1.107 (1.055, 1.161)	57746.06	0.330	0.176
PM_{10}	Main model	1.025 (0.987, 1.064)	57545.67	0.697	ref.
PM_{10}	Simplified model	1.041 (1.003, 1.081)	57758.29	0.310	0.540
NO_x	Main model	1.052 (1.009, 1.097)	57541.86	0.719	ref.
NO_x	Simplified model	1.087 (1.044, 1.131)	57747.36	0.340	0.268
NO_2	Main model	1.088 (1.032, 1.147)	57537.74	0.726	ref.
NO_2	Simplified model	1.132 (1.076, 1.192)	57741.10	0.350	0.283

Mixture	Main model	1.068 (1.018, 1.121)	57544.09	0.756	ref.
Mixture	Simplified model	1.106 (1.054, 1.160)	57747.96	0.480	0.322

HR: hazard ratio; CI: confidence interval. Simplified models were adjusted for age at recruitment, sex, ethnicity, and average time spent outdoors in summer and winter. Main models were adjusted for age at recruitment, sex, ethnicity, educational level, household income, BMI, employment status, time spent outdoors, and status of healthy lifestyle. AIC, Akaike Information Criterion. P_{ph}, the global p-value for the proportional hazard assumption test based on Schoenfeld residuals. P_{diff} denotes the p-value between-group differences in effect estimates.

References:

1. Jia J, Wang M, Shi Y, et al. Sleep health as a mediator between depression and functional gastrointestinal disorders: A UK Biobank study. *J Affect Disord* 2026; **395**(Pt B): 120768.
2. Chen J, Zhang H, Fu T, et al. Exposure to air pollution increases susceptibility to ulcerative colitis through epigenetic alterations in CXCR2 and MHC class III region. *eBioMedicine* 2024; **110**.
